# Giant Cyst of Dermis-Fat Graft in a Child with MRSA—Case Report

**DOI:** 10.3390/children12040457

**Published:** 2025-04-02

**Authors:** Biljana Kuzmanović Elabjer, Mirjana Bjeloš, Ana Ćurić, Daliborka Miletić, Mladen Bušić

**Affiliations:** 1 University Eye Department, Reference Center of the Ministry of Health of the Republic of Croatia for Pediatric Ophthalmology and Strabismus, Reference Center of the Ministry of Health of the Republic of Croatia for Inherited Retinal Dystrophies, Reference Center of the Ministry of Health of the Republic of Croatia for Standardized Echography in Ophthalmology, University Hospital “Sveti Duh”, Sveti Duh 64, 10000 Zagreb, Croatia; belabjer@kbsd.hr (B.K.E.); acuric@kbsd.hr (A.Ć.); dmiletic@kbsd.hr (D.M.); mbusic@kbsd.hr (M.B.); 2 Faculty of Medicine, Josip Juraj Strossmayer University of Osijek, Josipa Huttlera 4, 31000 Osijek, Croatia; 3 Faculty of Dental Medicine and Health Osijek, Josip Juraj Strossmayer University of Osijek, Crkvena 21, 31000 Osijek, Croatia

**Keywords:** retinoblastoma, exophthalmos, methicillin-resistant *Staphylococcus aureus*, cysts

## Abstract

Background/Objectives: This case report presents a unique case of multiple postoperative complications, including sterile silicone implant extrusion, symblepharon formation, and the development of a giant cyst, following extensive multimodal chemotherapy for unilateral retinoblastoma in a pediatric patient. The case was further complicated by recurrent methicillin-resistant *Staphylococcus aureus* (MRSA) colonization, which persisted despite multiple eradication attempts. Methods: A 5-year-old boy presented with right-sided proptosis one year after receiving a secondary dermis-fat orbital graft. He had undergone 12 cycles of intravitreal, intra-arterial, and systemic chemotherapy as well as thermotherapy and cryotherapy due to recurrent retinoblastoma in the right eye. Following a third relapse, secondary enucleation was performed with a primary silicone orbital implant. However, extrusion of the implant occurred, and an orbital swab confirmed MRSA colonization. A secondary dermis-fat graft was harvested and implanted after ensuring MRSA clearance. A year later, the child developed rapid right-sided proptosis. Ultrasound revealed a cyst within the dermis-fat graft measured 23.6 mm in anteroposterior diameter. Surgery was postponed due to chickenpox, and the cyst enlarged reaching an anteroposterior diameter of 26.7 mm over two months. A complete excision was performed. Results: The surgery was uneventful. Intraoperative orbital swab was sterile, but MRSA was detected in a conjunctival swab, leading to treatment with local moxifloxacin drops and oral rifampicin. Conclusions: Giant cyst formation in a dermis-fat graft is an extremely rare complication. Complete excision remains the treatment of choice. However, in this case, it resulted in persistent anophthalmic socket syndrome, posing further reconstructive challenges.

## 1. Introduction

Dermis-fat grafts (DFGs) have long been a cornerstone in orbital reconstructive surgery, offering a natural and biocompatible solution for restoring lost volume following enucleation or evisceration. These autologous grafts, composed of both dermal and adipose tissue, provide significant advantages, including seamless integration and a reduced risk of immune rejection. However, despite their benefits, complications can arise—most notably, cyst formation [1,2]. Typically lined with epithelial cells, these cysts can lead to clinical complications such as proptosis and discomfort [3]. While the reported incidence of cyst formation in anophthalmic sockets ranges from 1.5% to 3.8% [1], cases of extreme macrocystic enlargement remain exceedingly rare [2,3].

This report presents a case of giant cyst formation in a pediatric patient with recurrent retinoblastoma, illustrating the complex trajectory of orbital reconstruction. After enucleation and initial implantation of a silicone prosthesis, the patient faced a series of escalating complications, beginning with implant extrusion compounded by MRSA orbital colonization. This necessitated secondary reconstruction with a dermis-fat graft, yet further challenges emerged. Symblepharon developed postoperatively, requiring surgical correction with an amniotic membrane graft. Unknown at the time, an underlying cystic process was already underway—one that, within a month of symblepharon repair, progressed with alarming speed, culminating in profound proptosis. This case not only highlights the intricate and often unpredictable nature of orbital reconstruction but also underscores the delicate interplay of oncologic history, surgical intervention, and postoperative tissue healing dynamics.

## 2. Case Presentation

A 5-year-old male patient was diagnosed with hereditary retinoblastoma in the right eye (RE) in February 2022, classified as stage D according to the International Intraocular Retinoblastoma Classification system. Due to recurrent retinoblastoma in the RE, the patient underwent 12 treatment cycles, which included a combination of intravitreal injections, intra-arterial chemotherapy, systemic chemotherapy, thermotherapy, and cryotherapy.

### 2.1. Retinoblastoma Management

The treatment course began in March 2022, when the first cycle involved an intravitreal injection of melphalan in the RE, followed by systemic chemotherapy with etoposide and carboplatin. The second treatment cycle, administered in April 2022, consisted of intra-arterial chemotherapy with melphalan in the RE and an additional intravitreal injection of melphalan. In May 2022, the third treatment cycle was carried out with melphalan, administered both intravitreally and intra-arterially in the RE.

The fourth and fifth cycles, conducted in June 2022, included thermotherapy and cryotherapy.

The sixth treatment cycle, in August 2022, was managed also with thermotherapy and cryotherapy. In September 2022, the seventh cycle involved combined intra-arterial chemotherapy with melphalan and topotecan. The eighth cycle, in October 2022, also incorporated combined intra-arterial chemotherapy with melphalan and topotecan. The ninth cycle, administered in November 2022, involved thermotherapy of the RE. The tenth cycle, performed in February 2023, included ICG thermotherapy of the RE due to disease relapse. The eleventh and twelfth cycles, administered in March 2023 involved ICG thermotherapy of the RE, again due to relapse. In April 2023, examination revealed a parapapillary recurrence in the RE, with involvement of the optic nerve temporally. Given the resistance to ongoing treatment and evidence of tumor progression, enucleation was recommended as the safest option for the patient.

### 2.2. Primary Reconstruction and Implant Extrusion

Subsequent to the third relapse of retinoblastoma, secondary enucleation of the RE was performed in May 2023, with the placement of a primary silicone orbital implant. Histopathological analysis of the eyeball revealed the bulbus measuring 23:18:23 mm and the optic nerve section extending 15 mm in length and approximately 4–5 mm in width. Histologically, tumor tissue confirmed retinoblastoma without evidence of the optic nerve invasion. Six days postoperatively, implant extrusion occurred, requiring emmergent revision of the conjunctiva. Although no signs of infection were observed during the revision procedure, an orbital swab was obtained, and the conjunctiva was irrigated with a solution containing garamycin 80 mg/2 mL and dexamethasone 4 mg/mL. The culture of the orbital swab revealed colonization with Methicillin-resistant *Staphylococcus aureus* (MRSA).

In response to the MRSA colonization, a two-week course of intravenous vancomycin 200 mg q.i.d. and ceftriaxone 1 g q.d. was initiated. Additionally, topical azithromycin eye drops b.i.d. were applied for two weeks. A follow-up conjunctival swab confirmed successful eradication of the pathogen. As part of the planning for secondary reconstruction with a DFG in September 2023, an MRI of the brain and orbits was performed, confirming the absence of any retinoblastoma recurrence. During a routine preoperative ophthalmologic examination, despite the absence of clinical signs of conjunctival infection, a conjunctival swab was taken, which once again confirmed MRSA colonization. In response, an eradication regimen was initiated, consisting of moxifloxacin eye drops q.i.d. applied to the right orbit, along with oral rifampicin 300 mg q.d. for 7 days. Additionally, the regimen included preoperative chlorhexidine baths and the application of mupirocin to the nasal vestibule of both nostrils for five days prior to the procedure. A follow-up conjunctival swab confirmed no microbial presence.

### 2.3. Secondary Reconstruction

**Figure 1:**
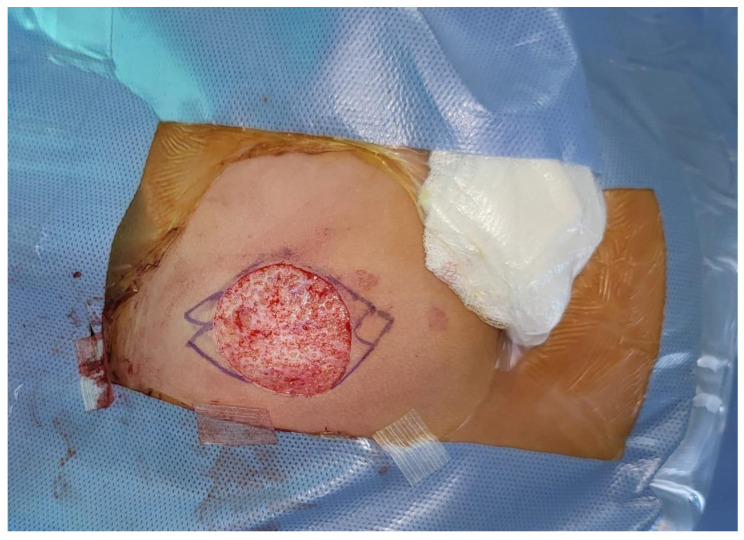
Dermis-fat graft was harvested from the right buttock, precisely midway between the ischial tuberosity and the ipsilateral greater trochanter.

Following verification of MRSA clearance, a secondary DFG was performed in November 2023. The graft was harvested by the pediatric plastic surgeon from the right buttock, precisely midway between the ischial tuberosity and the ipsilateral greater trochanter (Figure 1).

During graft preparation, the epidermis over the intended graft site was shaved using a No. 15 surgical blade, ensuring optimal tissue integrity. The underlying dermis and adipose tissue were excised via blunt dissection with scissors, separating fat lobules to minimize intraoperative bleeding. The harvested graft measured 25 mm in length, 20 mm in depth, and 20 mm in width. Closure of the donor site was achieved in two layers: the subcutaneous tissue was sutured with 4-0 resorbable sutures, while the skin was approximated using interrupted 4-0 non-resorbable sutures.

The conjunctiva was extended over the dermal portion of the graft by 2 mm and secured using interrupted 6-0 polyglycolate sutures, a procedure carried out by the oculoplastic surgeon. Special attention was given to prevent the burial of the conjunctival edges, as this is a recognized risk factor for cyst formation. As part of periprocedural prophylaxis, intravenous vancomycin 60 mg/kg was administered 60 min before the incision, with strict adherence to aseptic and antiseptic protocols. Conjunctival, nasal, throat, axillary, and groin swabs were collected for microbial surveillance.

Postoperatively, the patient received extended prophylactic vancomycin for 48 h at a dosage of 60 mg/kg/day, alongside local administration of azithromycin 1% ophthalmic drops (one drop b.i.d. for two days, followed by one drop q.d. for five days). The postoperative course was uneventful, with no pathogens isolated from conjunctival or systemic swabs, including conjunctival swabs obtained from the parents. The graft demonstrated successful integration, achieving complete epithelialization within six weeks, allowing for the subsequent fitting of an ocular prosthesis.

By May 2024, the patient exhibited discrete protrusion of the epiprosthesis, accompanied by significant shortening of the lower fornix due to temporal synechiae. The upper fornix was markedly shallow in its nasal third, with fusion of the palpebral and bulbar conjunctiva. To restore the fornices, surgical reconstruction with an amniotic membrane graft was performed in July 2024. A subsequent conjunctival swab identified MRSA colonization, albeit in low quantities. Targeted antimicrobial therapy was initiated with oral rifampicin and moxifloxacin ophthalmic drops, following the previously established regimen.

### 2.4. Giant Cyst Development

**Figure 2:**
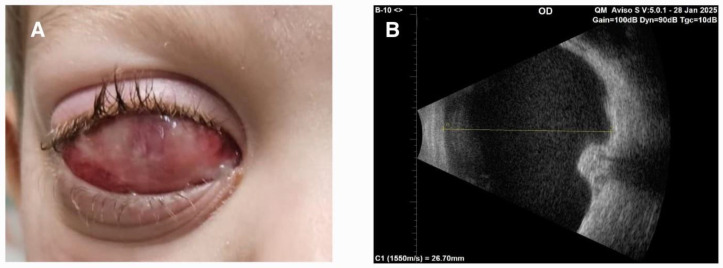
Photograph showing incomplete eyelid closure with upper eyelid retraction (**A**). Ultrasound B scan revealed cyst with anteroposterior diameter of 26.7 mm containing low-reflective fluid interspersed with fine, dot-like echoes (**B**).

In November 2024, the patient presented with rapidly progressive right-sided proptosis and significant eyelid enlargement. B-scan ultrasonography of the DFG revealed a well-demarcated cyst with a thin, highly reflective lining, measuring 23.6 mm in anteroposterior diameter. The cyst contained low-reflective fluid interspersed with fine, dot-like echoes. Due to concurrent varicella (chickenpox) infection, surgical intervention was deferred. Over the following two months, the child developed incomplete eyelid closure, with evident upper eyelid retraction (Figure 2A). The cyst exhibited further enlargement, ultimately reaching an anteroposterior diameter of 26.7 mm (Figure 2B).

**Figure 3:**
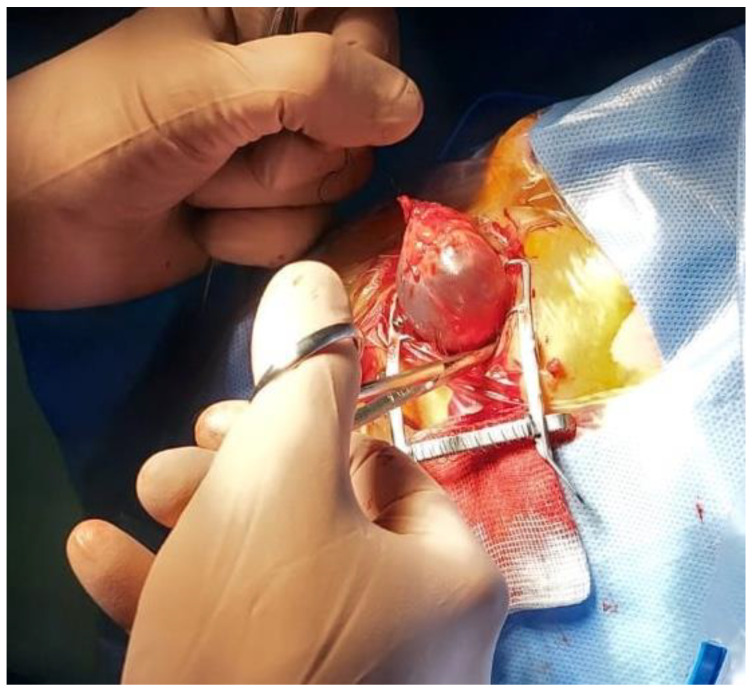
En bloc excision of the giant cyst.

Intraoperatively, the central dermal portion of the DFG was found to be firmly adherent to the cyst wall, necessitating an *en bloc* excision to mitigate the risk of recurrence (Figure 3).

**Figure 4:**
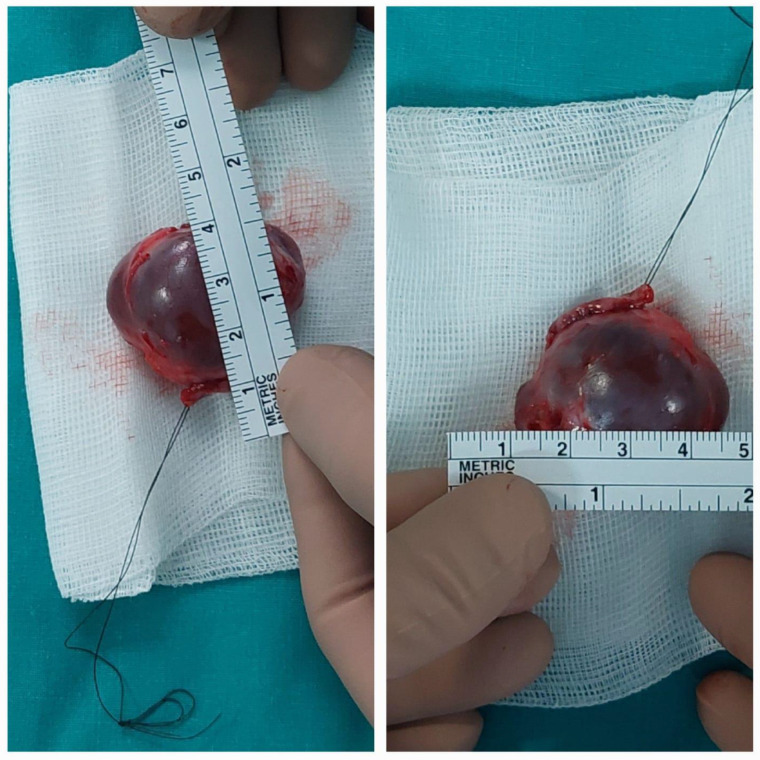
Photograph showing excised multilobulated fluid-filled giant cyst.

The excised lesion was macroscopically multilobulated, fluid-filled, and measured 4 × 3 × 3 cm (Figure 4).

The socket closure was performed with interrupted 6-0 resorbable sutures for Tenon’s capsule and running 8-0 sutures for the conjunctiva. A silicone conformer was placed intraoperatively to maintain socket integrity.

The cyst excision was uneventful, with sterile intraoperative orbital swabs. However, conjunctival swabs revealed low-level MRSA colonization, warranting a seven-day course of topical moxifloxacin q.i.d. and oral rifampicin 300 mg q.d. Histopathological examination was consistent with an epithelial inclusion cyst.

The patient has been under close monitoring for two months with no evidence of recurrence observed to date.

## 3. Discussion

### 3.1. Primary Reconstruction and Implant Extrusion

The extrusion of the implant in this case represents a complex interplay of treatment-related complications. The spontaneous loss of the silicone implant was attributed primarily to orbital tissue necrosis induced by intensive chemotherapy rather than to MRSA colonization. In this case, no signs of clinical infection suggested a limited capacity for active MRSA infection and a potential for spontaneous resolution. Chemotherapeutic agents exert cytotoxic effects on various cell types involved in tissue repair, exacerbating healing deficiencies, particularly in cases where chemotherapy is administered in conjunction with surgical interventions [4,5].

### 3.2. Secondary Reconstruction

The selection of donor adipose tissue significantly influences graft stability and susceptibility to resorption. Compared to abdominal fat, buttock fat demonstrates a more favorable fatty acid composition, characterized by higher monounsaturated fatty acids [6], which enhance resistance to oxidative stress and metabolic degradation. In contrast, abdominal fat, with its higher proportion of pro-inflammatory polyunsaturated fatty acids [6], is more prone to metabolic disturbances, increased inflammation, and accelerated resorption. These differences suggest that buttock-derived fat grafts may exhibit superior long-term viability, reduced susceptibility to necrosis, and improved integration. However, in adults, the abdominal region offers a more abundant fat layer with superior consistency for volume maintenance, making it a preferred choice in older patients.

### 3.3. Giant Cyst Development

While cyst formation is a known complication of DFGs [1,7,8], the emergence of a giant cyst is an exceedingly rare, but alarming event [2,3,9].

The development of giant cysts following dermis-fat grafting in enucleated patients represents a complex, multifactorial facet. A principal etiological factor is the inadvertent incorporation of residual epithelial cells due to incomplete excision of the conjunctival or corneal epithelium during enucleation [3]. These residual epithelial cells may become entrapped within the graft and subsequently proliferate, particularly within microscopic crevices between the recipient conjunctiva and the graft, ultimately leading to the formation of epithelial-lined cavities that expand into cystic structures over time [2]. The intrinsic properties of the graft further predispose it to cystogenesis, as the dermal component is susceptible to fibrosis and incomplete revascularization, thereby creating hypoxic microenvironments that disrupt normal tissue remodeling and promote pathological healing responses [10]. Furthermore, the adipose component of the graft is vulnerable to resorption and necrosis, leading to the formation of fluid-filled spaces that can serve as precursors to cyst formation [11]. A further contributory mechanism is the delayed conjunctivalization of the graft, which prolongs exposure of the underlying dermis to chronic irritation and inflammation, facilitating epithelial proliferation and possible migration into the deeper layers of the graft. The interaction of these pathological mechanisms, along with chemotherapeutic alterations to the healing process, leads to the gradual enlargement of cysts, emphasizing the need for strict preventive strategies, such as thorough epithelial debridement during surgery, improved graft preparation methods to reduce necrosis, and careful post-operative monitoring.

The striking inseparability of the overlying dermis from the anterior cyst wall hinted at a strong adhesion that likely contributed to cyst formation. Due to this firm connection, en bloc excision was necessary to prevent recurrence. Complete surgical excision remains the gold standard, as incomplete removal carries a substantial risk of recurrence.

Chemotherapeutic agents have long been suspected of contributing to cyst formation [12,13], though the link in this case remains unlikely.

### 3.4. MRSA Management

The management of asymptomatic MRSA colonization in a patient with a history of systemic chemotherapy and multiple surgical interventions presented a significant clinical challenge. While a low bacterial load may suggest a non-proliferative state, reducing the immediate risk of systemic infection, even minimal MRSA colonization can pose a serious threat in immunocompromised individuals. Although MRSA colonization without overt infection does not universally necessitate treatment, the unique circumstances of this case warranted a more cautious approach [14].

Given the patient’s extensive chemotherapy history, immune function was likely compromised, diminishing the body’s ability to control bacterial proliferation. This concern was particularly relevant in the context of orbital surgery, where the conjunctival surface maintains direct communication with deeper orbital tissues and, ultimately, the central nervous system. Such anatomical considerations elevate the potential risk of MRSA invasion into critical structures, increasing susceptibility to severe complications such as orbital cellulitis and meningitis [15,16,17].

Although the MRSA burden detected was low, the patient’s immunosuppressed state and history of complex surgical interventions justified an aggressive approach to colonization management, as detailed in the Case Presentation.

## 4. Conclusions

Giant cyst formation within a DFG represents an exceptionally rare complication, demanding precise surgical intervention. While complete excision remains the definitive treatment, this case underscores a stark reality—the persistence of an anophthalmic socket despite meticulous reconstructive efforts.

The complex interaction between the patient’s oncologic history, immune status, young age, and recurrent MRSA presence required a proactive and vigilant approach, incorporating evidence-based strategies to mitigate the risks of recurrent MRSA colonization. This case highlights the importance of meticulous surgical planning, rigorous infection control protocols, and comprehensive long-term follow-up in high-risk pediatric populations.

## Data Availability

The original contributions presented in this study are included in the article. Further inquiries can be directed to the corresponding author.
